# Biorenewable Oxypropylated Pentane-1,2,5-triol as a Source for Incorporation in Rigid Polyurethane Foams

**DOI:** 10.3390/polym15204148

**Published:** 2023-10-19

**Authors:** Georgy Grancharov, Mariya-Desislava Atanasova, Radostina Kalinova, Pencho Tuleshkov, Petar D. Petrov, Maya K. Marinova, Martin A. Ravutsov, Svilen P. Simeonov

**Affiliations:** 1 Institute of Polymers, Bulgarian Academy of Sciences, Acad. G. Bontchev Str. bl. 103A, 1113 Sofia, Bulgaria; m.atanasova@polymer.bas.bg (M.-D.A.); kalinova@polymer.bas.bg (R.K.); pen.tul@polymer.bas.bg (P.T.); ppetrov@polymer.bas.bg (P.D.P.); 2 Institute of Organic Chemistry with Centre of Phytochemistry, Bulgarian Academy of Sciences, Acad. G. Bontchev Str. bl. 9, 1113 Sofia, Bulgaria; maya.marinova@orgchm.bas.bg (M.K.M.); martin.ravutsov@orgchm.bas.bg (M.A.R.); svilen.simeonov@orgchm.bas.bg (S.P.S.); 3 Research Institute for Medicines (iMed.ULisboa), Faculty of Pharmacy, Universidade de Lisboa, Av. Prof. Gama Pinto, 1649-003 Lisbon, Portugal

**Keywords:** pentane-1,2,5-triol, polyurethanes, Achmatowicz rearrangement, biorenewable C5 alcohols, oxypropylation

## Abstract

In this study, as a product from the efficient Achmatowicz rearrangement and mild subsequent hydrogenation–reduction reactions of biorenewable C5 alcohols derived from lignocellulose, pentane-1,2,5-triol was successfully used after oxypropylation in the preparation of rigid polyurethane foams—one of the most important classes of polymeric materials. Despite the broad range of applications, the production of polyurethanes is still highly dependent on petrochemical materials considering the need of renewable raw materials and new process technologies for the production of polyol or isocyanate components as a key point for the sustainable development of polyurethane foams. The synthesized oxypropylated pentane-1,2,5-triol was analyzed using proton NMR spectroscopy, hydroxyl number, and viscosity, whereas the newly obtained foams incorporated with up to 30% biorenewable polyol were characterized using compressive stress, thermogravimetry, dynamic mechanical analysis, and scanning electron microscopy. The modified rigid polyurethanes showed better compressive strength (>400.0 kPa), a comparable thermal degradation range at 325–450 °C, and similar morphological properties to those of commercial polyurethane formulations.

## 1. Introduction

Nowadays, polyurethanes are a key class of polymeric materials and, among them, polyurethane foams (PUFs) correspond to 67% of the total polyurethane consumption [1,2]. PUFs show better thermal insulation properties than other commercially available insulating materials such as mineral wool or expanded polystyrene, making them the first choice for a wide range of applications such as automotive, electronics, furnishing, footwear, packaging, or construction materials [3,4,5,6]. Efforts of the polyurethane industry are currently focused on the replacement of petro-based feedstocks with bio-based ones [7,8,9,10,11,12]. 

The first attempts for a green and sustainable PUFs were the inclusion of polyols derived from natural sources, such as vegetable oils [13,14], agricultural wastes [15,16], and lignocellulosic biomass [17,18,19]. Different vegetable oils, such as castor oil [20,21], soybean oil [13,22], palm oil [23,24], rapeseed oil [25,26], tung oil [15,27], mustard seed oil [28,29], or canola oil [30,31], were used as precursors for the preparation of bio-polyols and the corresponding environmentally friendly bio-based PUFs [32,33]. Several studies have reported the preparation of bio-polyols or addition of post-agricultural products from corn stalks [34,35], cotton stalks [36], and wheat stalks [37,38,39] to develop PUF formulations. Among the bioresources utilized for the production of bio-based polyurethane foams, elastomers, coatings, and adhesives, lignin plays a key role, being the most abundant product on the Earth and a major component of lignocellulose [40,41,42]. Usually, for the preparation of the desired PUFs, lignin was used as an unmodified additive [43,44], liquefied by hydroxyl-containing compounds [45,46], or converted into bio-polyol via the oxypropylation process [47,48,49]. The latter case comprises the use of a suitable catalyst, temperature, and pressure during the course of the oxypropylation [50]. Valuable polyurethane structures were obtained on the basis of bio-renewable polyols such as self-healing and antibacterial materials, as well as controlled-release fertilizers [51]. In order to obtain or improve key parameters of bio-based PUFs, some inorganic filling materials such as nano-silica, aluminum silicate, and ferroso-ferric oxide nanoparticles were added into polyurethane formulations to enhance their physical–mechanical [52], dielectric [53], and magnetic properties [54], respectively.

The other major lignocellulose component, hemicellulose, possesses an abundance of xylans and is mainly composed by pentoses, which are used in the production of furfural. Furfural is defined as an easily accessible, cheap, and important bio-based compound, finding application in the synthesis of different chemicals, but its main part during production is usually hydrogenated to the C5 alcohol form—furfuryl alcohol [55,56]. The C5 alcohols are important materials for the industry assuming their application in polyurethane, polyester, polyether, and fuel additive production [57], and they were synthesized via hydrolysis or hydrogenolysis of the furan ring in very harsh conditions [58,59,60]. The synthesis of pentane-1,2,5-triol (125PTO) is not well studied, since Simeonov et al. reported a high-yielding preparation of 125PTO from furfuryl alcohol [61,62]. Here, the catalytically challenging reactions have been avoided using the Achmatowicz rearrangement product and easily accessible catalysts in mild conditions. Initially, furfuryl alcohol was converted into 6-hydroxy-(2H)-pyran-3(6H)-one (Achmatowicz intermediate) via the Achmatowicz rearrangement reaction in the presence of titanium silicalite, and next, 125PTO was obtained after Pd/C hydrogenation and NaBH_4_ reduction of the Achmatowicz intermediate. Furthermore, a flow chemistry synthesis based on Ru/C-catalyzed hydrogenation has also been reported [61]. Alternatively, 125PTO can be prepared via the gas-phase hydrogenation reaction catalyzed by modified Ni and/or Pt mesoporous silica catalysts. The conversion and selectivity of the newly prepared catalysts reached approximately 100% based on the intermediate product [62].

In the present study, the preparation of the oxypropylated biorenewable pentane-1,2,5-triol obtained via Achmatowicz rearrangement and batch reduction reactions was investigated. The successful inclusion of that bio-based 125PTO polyol into new polyurethane formulations at quantities up to 30%, and its subsequent influence on the physical–mechanical, thermal, and morphological properties of modified rigid PUFs was also reported.

## 2. Experiment 

### 2.1. Materials

Catalyst titanium silicalite was synthesized as described previously with some modifications [61]. Rigid PUF insulating system “Elastopor” (BASF) was used for PUF preparation. It includes polyol A component containing polyether polyol, catalyst, surfactant, foam agent, and isocyanate B component consisting of polymeric diphenylmethane 4, 4′-diisocyanate (pMDI—31 wt% of isocyanate groups). Furfuryl alcohol, sodium borohydride (NaBH_4_), propylene oxide (PrO), pyridine, acetic anhydride, potassium hydroxide, potassium carbonate, magnesium sulfate, hydrochloric acid, acetonitrile, ethyl acetate, hexane, methylene chloride, ethanol, methanol, and isopropanol (Sigma Aldrich–Merck, Darmstadt, Germany) were used as received. 

### 2.2. Instruments and Methods

^1^H NMR spectra were recorded on a Bruker Avance II apparatus operating at 600 MHz. For the measurements, 12 mg of sample were dissolved in 0.7 mL deuterated solvents (DMSO-d_6_, and CDCl_3_). The viscosity of samples was measured on a Rheometer RheoStress 600 (Thermo Haake, Waltham, MA, USA) using 500 µL drops at 25 °C. The compressive strength and the compressive modulus of elasticity were tested at a 5 mm/min displacement rate of the compression plate on a “TIRA TEST 2300” (Schalkau, Germany) testing machine using ISO 844 [63]. The maximum force inducing a 10% relative strain was determined by decreasing the foam height in relation to the initial height according to the direction of foam growth on 5 × 5 × 5 cm cubes. Thermogravimetric analyses were performed on a TGA4000 (PerkinElmer, Shelton, CT, USA) combined with a gas chromatograph and mass-selective detector. The equipment is supplied with PYRIS6 software (PerkinElmer, Shelton, CT, USA) measuring mass change in inert gas flow (argon) with a controlled-temperature elevation of approximately 10 mg samples. Dynamic Mechanical Analyzer-Q800 (TA Instruments, New Castle, DE, USA) was used for determining the viscoelastic properties of PUFs as a function of temperature on bar samples with dimensions 25 × 5 × 5 mm. The foam structure was analyzed using scanning electron microscope (SEM) Philips 515 (Eindhoven, The Netherlands) with secondary electron image detectors (SEI), acceleration voltage 30 kV, magnification 40,000×, and 5 × 5 mm thin sheets. The hydroxyl number (OH no) of samples was determined using an acylation method with acetic anhydride in pyridine as the medium. An excess of acetic anhydride after hydrolysis and the obtained acetic acid was titrated by standard potassium hydroxide solution and phenolphthalein as an indicator. The foaming process was analyzed in accordance with ASTM D7487-13e [64] through implementation of the PUF cup test and measuring PUF’s cream, gel, and tack-free times. The apparent density of foams (the ratio of foam weight to its geometrical volume) was determined for cube-shaped samples with a side length of 50 mm in accordance with ISO 845 [65]. The closed cell content and the water absorption of the obtained PUFs were determined in accordance with standard procedures ISO 4590 and ISO 2896, respectively [66,67]. Dimensional stability of the foams was carried out in the thermostating process of samples at temperatures −25 °C and 100 °C in 48 h. The result of this test included a change in linear dimensions of PUFs in accordance with ISO 2796 [68]. The bio-based mass content of the PUFs was calculated as a percentage of the total mass of samples available in accordance with ISO 16620-4 [69].

### 2.3. Synthesis of 6-Hydroxy-(2H)-pyran-3(6H)-one

Furfuryl alcohol (20.0 g), distilled before experiment, was dissolved in acetonitrile (200 mL) and then the catalyst TS-1 (2.0 g) was slowly added to the solution. It was followed by dropwise addition of aq. H_2_O_2_ (37%, 30 mL), and the reaction mixture was heated and stirred at 40 °C for 5 h. The reaction progress was monitored through thin layer chromatography (EtOAc/Hexane = 1:1) following the full consumption of furfuryl alcohol. The crude mixture was filtered and the obtained filtrate was evaporated. Next, it was dissolved in methylene chloride and dried over magnesium sulfate to give 6-hydroxy-(2H)-pyran-3(6H)-one (21.6 g, 94% yield), without the need of further purification. The product was obtained as pale-yellow oil that crystallizes at −12 °C.

^1^H NMR (600 MHz, CDCl_3_): d = 6.98 (dd, 1H), 6.18 (d, 1H), 5.64 (d, 1H), 4.58 (d, 1H), 4.15 (d, 1H), 3.80 ppm (s, 1H).

### 2.4. Synthesis of Pentane-1,2,5-triol

A mixture containing 6-hydroxy-(2H)-pyran-3(6H)-one (12.6 g, 0.11 mol), ethanol (280 mL), and catalyst Pd/C (1.3 g, 10 wt%) was allowed to react in H_2_ atmosphere at room temperature for 5 h. The obtained mixture was then filtered through a layer of Celite^®^ and the solvent was evaporated to yield 12.0 g of pale-yellow oil. The crude product was dissolved in methanol (200 mL) and NaBH_4_ (11.7 g, 0.31 mol) was slowly added in portions at 0 °C. The reaction mixture was warmed to RT and stirred for 24 h. After that, a prepared solution of i-PrOH/HCl was slowly added to the reaction suspension up to pH = 2 in order to decompose the boronic complexes. This was followed by addition of potassium carbonate (saturated solution in MeOH) to neutralize the added acid. The mixture was filtered through a paper filter and nylon membrane filter (pore size 0.45 µm, diameter 47 mm), followed by solvent evaporation and preparation of the desired product as oil (12.1 g, 97% yield).

^1^H NMR (400 MHz, DMSO-d_6_): d = 3.44–3.34 (m, 3H), 3.29–3.22 (m, 2H), 1.56–1.47 (m, 1H), 1.47–1.35 ppm (m, 2H), 1.24–1.15 ppm (m, 1H); 

### 2.5. Treatment of Pentane-1,2,5-triol by Propylene Oxide

Equipped with a magnetic stirrer, thermometer, reflux condenser, and dropping funnel, 4.3 g of biorenewable pentane-1,2,5-triol and 0.13 g of potassium hydroxide (~3.0% by weight) were charged into a glass reactor as a catalyst. The reaction system was heated at 120 °C and propylene oxide was charged into the dropping funnel. The temperature increased to 130 °C and, at this point, PrO (~8.0 mL) was added dropwise until the reaction temperature started to decrease, and the addition of propylene oxide was stopped. The reaction mixture was stirred additionally at 130 °C for 4 h and finally was subjected to vacuum drying (10 mm Hg, 110–115 °C) until a constant mass of the oxypropylated polyol (10.2 g). 

^1^H NMR (400 MHz, DMSO-d_6_), d = 5.12–4.80 (m, 3.6H), 3.80–3.10 (m, 13.4H), 1.85–1.18 (m, 4H), 1.04–0.92 ppm (m, 10.9H); 

### 2.6. Preparation of Rigid Polyurethane Foams

The rigid PUFs were prepared by mixing polyol A component and isocyanate B component of the rigid PUF insulating system at a weight ratio 1:1.2 (isocyanate index = 110). After intensive stirring for 10 s, the mixture was poured into a mold of dimensions 180/130/130 mm. Additionally, to initial polyurethane formulation (F-1) without biorenewable polyol, three types of rigid PUFs were prepared in this study, incorporating 10 wt% (F-2), 20 wt% (F-3), and 30 wt% (F-4) of biorenewable polyol through partial replacement of standard petrochemical polyol. Obtained PUFs were conditioned for 24 h at normal conditions after removal from the mold and cut to appropriate size for the different characterizations.

## 3. Results and Discussion

**Scheme 1:**
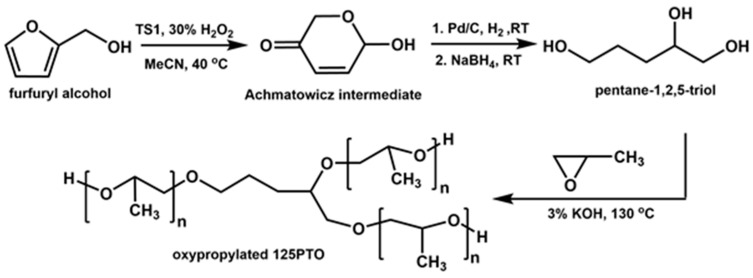
Synthesis of bio-based polyol from biorenewable hemicellulose product furfuryl alcohol.

Furfuryl alcohol, the hemicellulose-based product, was chosen as a starting compound for efficient preparation of pentane-1,2,5-triol. First, it was converted via Achmatowicz rearrangement to Achmatowicz intermediate, 6-hydroxy-(2H)-pyran-3(6H)-one, and the latter yielded the desired 125PTO after hydrogenation via mild batch reduction reaction. The synthesis of 6-hydroxy-(2H)-pyran-3(6H)-one via Achmatowicz rearrangement from furfuryl alcohol was achieved in 94% conversion using the titanium silicalite (TS-1) catalyst and 30% hydrogen peroxide system in acetonitrile at 40 °C (Scheme 1). Next, the conversion of the Achmatowicz intermediate to pentane-1,2,5-triol was performed via batch hydrogenation induced by the Pd/C catalyst, and immediate mild NaBH_4_ reduction at room temperature resulted in a 97% conversion (Scheme 1). Oxypropylation of the thus-prepared pentane-1,2,5-triol with propylene oxide was obtained in the presence of KOH as the catalyst at 130 °C, yielding bio-based polyol. It was found that pentane-1,2,5-triol and propylene oxide reacted at a ratio of 1:3.0 measured gravimetrically and 1:3.6 determined by ^1^H NMR data, and therefore, all three hydroxyl groups of 125PTO reacted at least to one equivalent of PrO (Scheme 1).

**Figure 1:**
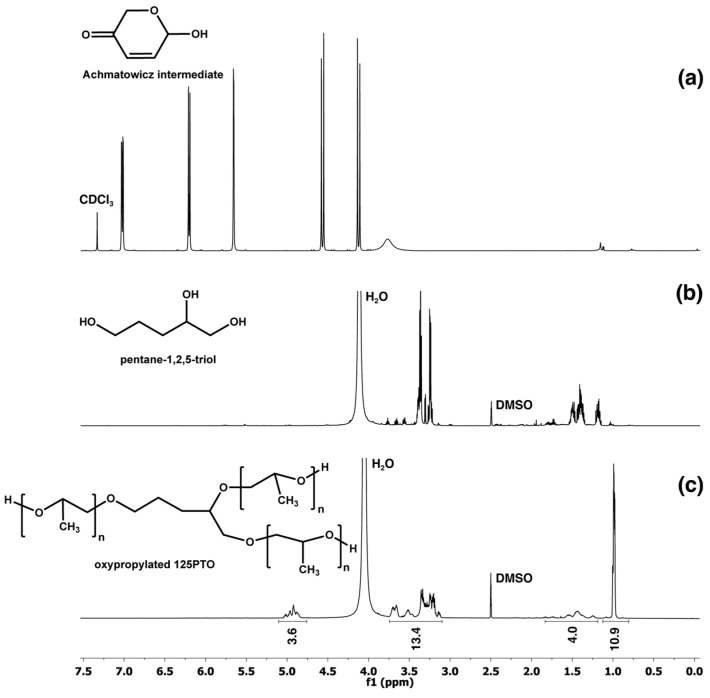
^1^H NMR spectrum of the products synthesized during preparation of biorenewable oxypropylated pentane-1,2,5-triol: (**a**) 6-hydroxy-(2H)-pyran-3(6H)-one (Achmatowicz intermediate), (**b**) pentane-1,2,5-triol, and (**c**) oxypropylated pentane-1,2,5-triol.

The three-step preparation of oxypropylated 125PTO achieved on the basis of biorenewable furfuryl alcohol was followed by ^1^H NMR spectroscopy. Firstly, hemicellulose derivative C5 furfuryl alcohol was reacted with the titanium silicalite catalyst—a 30% hydrogen peroxide system—in a rearrangement reaction, leading to the Achmatowicz intermediate possessing the ^1^H NMR spectrum shown in Figure 1a. The high yield conversion of the furfuryl alcohol precursor into the Achmatowicz rearrangement intermediate was confirmed through ^1^H NMR spectroscopy, revealing sharp signals assigned to protons of the cyclic product containing alkene—6.98 ppm (dd, 1H) and 6.18 ppm (d, 1H); methine—5.64 (d, 1H); methylene—4.58 ppm (d, 1H) and 4.15 ppm (d, 1H); and hydroxyl protons—3.80 ppm (s, 1H). In the second step, an approximately quantitative conversion of the Achmatowicz intermediate to pentane-1,2,5-triol with the ^1^H NMR spectrum presented in Figure 1b was achieved via combined batch hydrogenation induced by the Pd/C catalyst and immediate mild NaBH_4_ reduction. ^1^H NMR spectroscopy data show signals assigned to protons of linear product 125PTO—3.44–3.34 (m, 3H, for -OCH- and -OCH_2_-), 3.29–3.22 (m, 2H, for -OCH_2_-), 1.56–1.47 (m, 1H, for -CH_2_-), 1.47–1.35 ppm (m, 2H, for -CH_2_-), and 1.24–1.15 ppm (m, 1H, for -CH_2_-). The third step completes the reaction of 125PTO with propylene oxide in the presence of potassium hydroxide as the catalyst. The successful preparation of oxypropylated pentane-1,2,5-triol was also attested via ^1^H-NMR analysis (Figure 1c) to show signals of protons at 5.12–4.80 (m, 3.6H, for -OCH-), 3.80–3.10 (m, 13.4H, for -OCH- and -OCH_2_-), 1.85–1.18 (m, 4H, for -CH_2_-), and 1.04–0.92 ppm (m, 10.9H, for -CH_3_-).

The properties of the hydroxyl products related to PUF formulations are shown in Table 1.

The treatment of pentane-1,2,5-triol by propylene oxide reduces the hydroxyl number to 410 mg KOH/g and increases the viscosity to 180 mPa·s of bio-based polyol. This helps the newly obtained 125PTO polyol to approach properties similar to those of the initial polyol component used for the preparation of rigid PUFs and, in addition, to fulfil the requirements that a given polyol should possess when used in rigid polyurethane foam formulations—a hydroxyl number between 300 and 800, and a viscosity below 300 Pa·s [47,48].

**Scheme 2:**
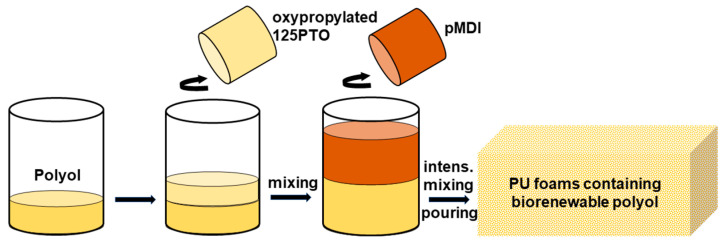
Preparation of rigid polyurethane foams containing biorenewable oxypropylated pentane-1,2,5-triol.

The renewable 125PTO-based polyol was introduced into the rigid PUF compositions in quantities up to 30 wt%, replacing the original system’s polyol component. Initially, the oxypropylated product was added to the polyol A component of the polyurethane system and thoroughly mixed. Next, an isocyanate-containing B component of the rigid PUF insulating system was included to such a prepared polyol mixture. After short intensive stirring, the whole mixture was poured into a mold where polyurethane foam started to rise and form (Scheme 2). 

The investigation of the reactivity of the new insulating PUFs shows that the cream time, gel time, and tack-free time are similar to those of the standard system (Table 2). The density of the new rigid PUFs increases with the amount of added 125PTO polyol, whereas the percentage of the closed cell content decreases with the quantity of incorporated biorenewable polyol (Table 2). The bio-based mass content of rigid PUFs with 10, 20, and 30% of added bio-based polyol is enhanced to 1.95, 3.90, and 5.85%, respectively (Table 2). 

**Figure 2:**
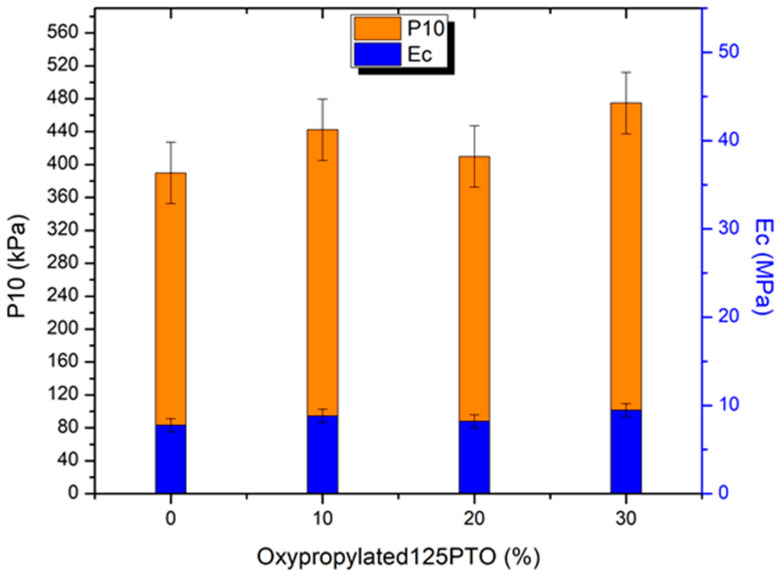
Influence of the content of oxypropylated renewable product on the compressive strength and the modulus of elasticity at compressive strength of polyurethane foams.

The presence of the oxypropylated bio-based product in the rigid polyurethane compositions causes a favorable effect on their physical–mechanical properties as well. The compressive strength at 10% relative deformation increases from 389.9 kPa for the standard composition (F-1) to 442.4, 409.9, and 474.9 kPa for rigid PUFs with 10, 20, and 30% of added bio-based polyol (F-2, F-3, and F-4), respectively, as shown in Figure 2. A similar relationship can be observed for the modulus of elasticity at compressive strength. It increases from 7.78 MPa for composition F-1 to 8.83, 8.21, and 9.47 MPa for compositions F-2, F-3, and F-4, respectively (Figure 2). The obtained results show that biorenewable 125PTO polyol can be successfully implemented in the preparation of new rigid PUF insulations with improved exploitation characteristics such as higher density and better mechanical properties.

An investigation of dimensional stability of rigid PUFs shows that polyurethane foams containing oxypropylated 125PTO (F-2, F-3, and F-4) possess identical and even better dimensional stability to the standard polyurethane composition F-1 determined at −25 °C and 100 °C for 48 h (Table 3). 

**Figure 3:**
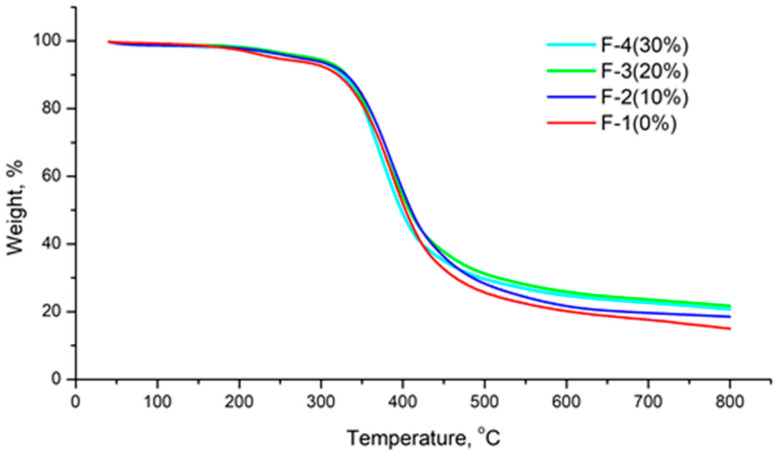
Thermogravimetric analyses of rigid polyurethane foams with added bio-based polyol (F-2, blue line; F-3, green line; and F-4, cyan line) and standard polyol component (F-1, red line).

Thermal degradation properties of PUFs were studied through thermogravimetric analyses (TGAs). The TGA data in Figure 3 depict that polyurethane foams with added bio-based polyol (F-2, F-3, and F-4), and the standard polyurethane foam (F-1) possess no significant difference in their thermal stability. The first weight loss at about 5% can be observed between 175 and 250 °C. It corresponds to the evaporation of low-molecular-weight products such as water, blowing agent, and some monomers. Most of the PUFs’ chemical bonds have not begun to break up in this stage. The major decomposition step for all samples indicating weight loss at about 75% starts at 275 °C and finishes at 475 °C. Here, the first stage at 275–375 °C is mainly connected with the initial degradation of urethane, urea, and isocyanurate bonds [70]. In the second degradation stage at 375–475 °C, the decomposition of urethane bonds continues and accomplishes a dissociation of polyol segments [71,72]. The char residue at the end of the degradation process is 15.0% for standard polyurethane composition F-1, whereas it is 18.5, 21.7, and 20.7% for those modified with oxypropylated polyol compositions F-2, F-3, and F-4, respectively. 

**Figure 4:**
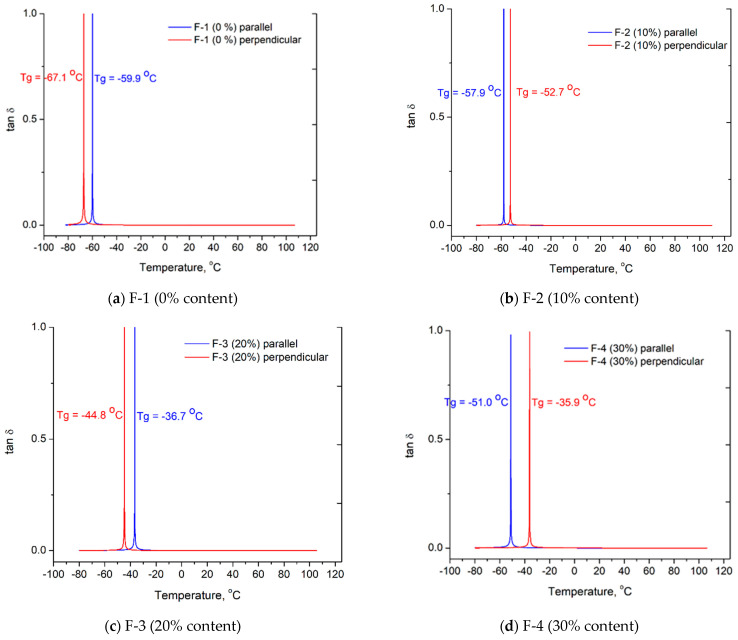
Dynamic mechanical analysis showing tan δ of rigid polyurethane foams with different contents of oxypropylated biorenewable product determined parallel and perpendicular to foam growth direction.

Further investigation of the thermal properties of PUFs was achieved through dynamic mechanical analysis (DMA), which measures the response of a given material to a cyclic deformation as a function of temperature. Usually, DMA results exhibit three main parameters: (i) the storage modulus, showing the elastic response to the deformation; (ii) the loss modulus, yielding the plastic response to the deformation; and (iii) tan δ, which is the loss-to-storage-modulus ratio, useful for the determination of occurrence of molecular mobility transitions known as the glass transition temperature (Tg). The sample with 10% of biorenewable 125PTO-based polyol shows an average Tg of −57.9 °C and −52.7 °C, whereas the foam with standard polyol exhibits a Tg of −59.9 °C and −67.1 °C, parallel and perpendicular to the growth direction, respectively (Figure 4). The increment in Tg was also observed for the sample with 20% of biorenewable polyol, showing an average Tg of −36.7 °C and −44.8 °C, and for foam with 30% of bio-based polyol, representing an average Tg of −51.0 °C and −35.9 °C, parallel and perpendicular to the growth direction, respectively. Obviously, an increase in the glass transition temperature for modified samples F-2, F-3, and F-4 in both foam growth directions can be seen in the investigated temperature range compared with standard sample F-1. This also indicates a possible increase in branching points, some restricted segmental motions, and a slight decrease in the elastic properties of the modified samples containing renewable polyol [72].

**Figure 5:**
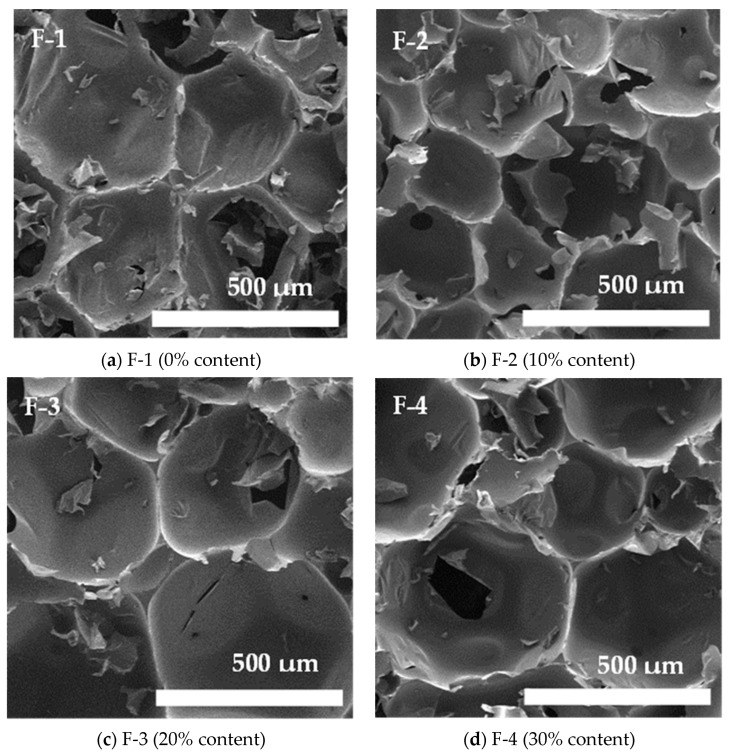
SEM analysis of rigid polyurethane foams prepared with different contents of oxypropylated bio-based product.

The morphological properties of those modified with 125PTO-based polyol PUFs were analyzed using scanning electron microscopy. PUF samples for SEM observation were cut from the perpendicular orientation to the foam growth direction. It can be seen from Figure 5 that the cell size of standard foam (F-1) and the foam with 10% of added biorenewable polyol (F-2) is smaller than that of the foams with 20 and 30% contents of bio-based polyol (F-3 and F-4), respectively. The cell size of foams with a lower content of 125PTO-based polyol varies in the range of 350 ± 50 µm, whereas the cell size of foams with a higher amount of added oxypropylated product is altered in the range of 480 ± 80 µm. Therefore, the use of oxypropylated 125PTO in quantities up to 30% is beneficial for the enhancement in cell size, i.e., thermal insulation ability of PUFs improves even the slight increase in their apparent foam density.

## 4. Conclusions

Biorenewable oxypropylated pentane-1,2,5-triol was synthesized from furfuryl alcohol via Achmatowicz rearrangement, subsequent batch hydrogenation–reduction reactions, and the terminative oxypropylation process. Bio-based polyol was successively introduced in new rigid polyurethane foam compositions at amounts up to 30% in place of standard polyol. The obtained polyurethane foams containing renewable polyol attained better compressive strength (>400.0 kPa), a comparable thermal degradation range at 325–450 °C, and similar morphological properties to those of the initial petro-based commercial polyurethane formulation. The modified rigid foams can be successfully implemented in the preparation of construction, industrial, and household insulations.

## Figures and Tables

**Table 1:** Properties of hydroxyl products related to rigid polyurethane foam system.

Property	Pentane-1,2,5-triol	Oxypropylated 125PTO	Polyol Component
Viscosity (mPa·s)	60	180	430
OH no. (mg KOH/g)	1380 *	410	300

* Theoretically calculated value.

**Table 2:** Characteristic data, reaction times, and properties of obtained rigid polyurethane foams.

Characteristic Data	F-1 (0%)	F-2 (10%)	F-3 (20%)	F-4 (30%)
Polyol (g)	10.0	9.0	8.0	7.0
pMDI (g)	12.0	12.0	12.0	12.0
Oxypropylated 125PTO (g)	0	1.0	2.0	3.0
wt (%) (based on polyol component)	0	10	20	30
wt (%) bio-based content (based on PU foam)	0	1.95	3.90	5.85
Reaction time at 25 °C cup test				
- Cream time (s)	20.2 ± 0.4	19.7 ± 0.4	19.5 ± 0.3	19.7 ± 0.3
- Gel time (s)	133.8 ± 2.5	119.1 ± 2.3	112.8 ± 2.2	116.5 ± 2.1
- Tack-free time (s)	185.6 ± 2.6	203.5 ± 2.8	198.7 ± 2.7	186.8 ± 2.5
Apparent density (kg/m^3^)	52.8 ± 1.5	56.0 ± 1.7	59.1 ± 1.8	64.3 ± 2.0
Closed cell content (%)	97.4 ± 1.3	97.2 ± 1.4	93.0 ± 1.4	90.4 ± 1.5

**Table 3:** Dimensional stability of rigid polyurethane foams.

Dimensional Stability (%)	F1 (0%)	F2 (10%)	F3 (20%)	F4 (30%)
48 h, −25 °C				
- Length (%)	2.1 ± 0.4	1.8 ± 0.3	1.1 ± 0.3	0.0 ± 0.0
- Width (%)	2.2 ± 0.5	0.0 ± 0.0	0.9 ± 0.2	0.0 ± 0.0
- Thickness (%)	2.9 ± 0.8	0.9 ± 0.2	1.0 ± 0.3	0.0 ± 0.0
48 h, 100 °C				
- Length (%)	−2.1 ± 0.3	−0.9 ± 0.2	0.0 ± 0.0	−0.4 ± 0.1
- Width (%)	2.6 ± 0.5	1.7 ± 0.4	1.8 ± 0.4	0.6 ± 0.2
- Thickness (%)	0.3 ± 0.1	0.7 ± 0.2	−0.7 ± 0.2	0.0 ± 0.0

## Data Availability

The data presented in this study are available on request from the corresponding author.

## References

[1] Ionescu M. (2005). Chemistry and Technology of Polyols for Polyurethanes.

[2] Szycher M. (2006). Szycher’s Handbook of Polyurethanes.

[3] Gama N.V., Ferreira A., Barros-Timmons A. (2018). Polyurethane foams: Past, present, and future. Materials.

[4] Eaves D. (2004). Handbook of Polymer Foams.

[5] Das S., Heasman P., Ben T., Qiu S. (2017). Porous organic materials: Strategic design and structure–function correlation. Chem. Rev..

[6] Ashida K. (2007). Polyurethane and Related Foams: Chemistry and Technology.

[7] Simón D., Borreguero A.M., de Lucas A., Rodríguez J.F. (2018). Recycling of polyurethanes from laboratory to industry, a journey towards the sustainability. Waste Manag..

[8] Furtwengler P., Avérous L. (2018). Renewable polyols for advanced polyurethane foams from diverse biomass resources. Polym. Chem..

[9] Chung H., Washburn N.R. (2013). Chemistry of lignin-based materials. Green Mater..

[10] Kazzaz A.E., Feizi Z.H., Fatehi P. (2019). Grafting strategies for hydroxy groups of lignin for producing materials. Green Chem..

[11] Ma Y., Xiao Y., Zhao Y., Bei Y., Hu L., Zhou Y., Jia P. (2022). Biomass based polyols and biomass based polyurethane materials as a route towards sustainability. React. Funct. Polym..

[12] Kaur R., Singh P., Tanwar S., Varshney G., Yadav S. (2022). Assessment of bio-based polyurethanes: Perspective on applications and bio-degradation. Macromol.

[13] Tan S., Abraham T., Ference D., Macosko C.W. (2011). Rigid polyurethane foams from a soybean oil-based polyol. Polymer.

[14] Brenes-Granados D., Cubero-Sesin J.M., Gutiérrez F.O., Vega-Baudrit J., Gonzalez-Paz R. (2017). Variation of physical properties of rigid polyurethane foams synthesized from renewable sources with different commercial catalysts. J. Renew. Mat..

[15] Da Silva V.R., Mosiewicki M.A., Yoshida M.I., Da Silva M.C., Stefani P.M., Marcovich N.E. (2013). Polyurethane foams based on modified tung oil and reinforced with rice husk ash I: Synthesis and physical chemical characterization. Polym. Test..

[16] Czlonka S., Bertino M.F., Strzelec K. (2018). Rigid polyurethane foams reinforced with industrial potato protein. Polym. Test..

[17] Li Y., Ren H., Ragauskas A.J. (2011). Rigid polyurethane foam/cellulose whisker nanocomposites: Preparation, characterization, and properties. J. Nanosci. Nanotechnol..

[18] Li Y., Ragauskas A.J. (2012). Kraft lignin-based rigid polyurethane foam. J. Wood Chem. Technol..

[19] Saffar T., Bouafif H., Braghiroli F.L., Magdouli S., Langlois A., Koubaa A. (2020). Production of bio-based polyol from oxypropylated pyrolytic lignin for rigid polyurethane foam application. Waste Biomass Valorization.

[20] Tavares L.B., Boas C.V., Schleder G.R., Nacas A.M., Rosa D.S., Santos D.J. (2016). Bio-based polyurethane prepared from Kraft lignin and modified castor oil. Express Polym. Lett..

[21] Ionescu M., Radojcic D., Wan X., Shrestha M.L., Petrovic Z.S., Upshaw T.A. (2016). Highly functional polyols from castor oil for rigid polyurethanes. Eur. Polym. J..

[22] Miao S., Sun L., Wang P., Liu R., Su Z., Zhang S. (2012). Soybean oil-based polyurethane networks as candidat biomaterials: Synthesis and biocompatibility. Eur. J. Lipid Sci. Technol..

[23] Adnan S., Tuan Noor M.T.I., ‘Ain N.H., Devi K.P.P., Mohd N.S., Kian Y.S., Idris Z.B., Campara I., Schiffman C.M., Pietrzyk K. (2017). Impact of the hard-segment concentration on highly resilient polyurethane foams based on palm olein polyol. J. Appl. Polym. Sci..

[24] Zhou X., Sain M.M., Oksman K. (2016). Semi-rigid biopolyurethane foams based on palm-oil polyol and reinforced with cellulose nanocrystals. Compos. Part A.

[25] Kairyte A., Vejelis S. (2015). Evaluation of forming mixture composition impact on properties of water blown rigid polyurethane (PUR) foam from rapeseed oil polyol. Ind. Crops Prod..

[26] Prociak A., Kuranska M., Cabulis U., Kirpluks M. (2017). Rapeseed oil as main component in synthesis of bio-polyurethane-polyisocyanurate porous materials modified with carbon fibers. Polym. Test..

[27] Da Silva V.R., Mosiewicki M.A., Yoshida M.I., Da Silva M.C., Stefani P.M., Marcovich N.E. (2013). Polyurethane foams based on modified tung oil and reinforced with rice husk ash II: Mechanical characterization. Polym. Test..

[28] Borowicz M., Paciorek-Sadowska J., Lubczak J., Czuprynski B. (2019). Biodegradable, flame-retardant, and bio-based rigid polyurethane/polyisocyanurate foams for thermal insulation application. Polymers.

[29] Paciorek-Sadowska J., Borowicz M., Czupryński B., Tomaszewska E., Liszkowska J. (2018). New bio-polyol based on white mustard seed oil for rigid PUR-PIR foams. Pol. J. Chem. Technol..

[30] Kong X., Liu G., Curtis J.M. (2012). Novel polyurethane produced from canola oil based poly(ether ester) polyols: Synthesis, characterization and properties. Eur. Polym. J..

[31] Karimi M.B., Khanbabaei G., Sadeghi G.M.M. (2019). Unsaturated canola oil-based polyol as effective nucleating agent for polyurethane hard segments. J. Polym. Res..

[32] Miao S., Wang P., Su Z., Zhang S. (2014). Vegetable-oil-based polymers as future polymeric biomaterials. Acta Biomater..

[33] Czlonka S., Strakowska A., Strzelec K., Kairyte A., Kremensas A. (2020). Bio-based polyurethane composite foams with improved mechanical, thermal, and antibacterial properties. Materials.

[34] Yan Y., Pang H., Yang X., Zhang R., Liao B. (2008). Preparation and characterization of water-blown polyurethane foams from liquefied cornstalk polyol. J. Appl. Polym. Sci..

[35] Hu S., Li Y. (2014). Two-step sequential liquefaction of lignocellulosic biomass by crude glycerol for the production of polyols and polyurethane foams. Bioresour. Technol..

[36] Wang Q., Tuohedi N. (2020). Polyurethane foams and bio-polyols from liquefied cotton stalk agricultural waste. Sustainability.

[37] Li H., Feng S., Yuan Z., Wei Q., Xu C. (2017). Highly efficient liquefaction of wheat straw for the production of bio-polyols and bio-based polyurethane foams. Ind. Crops Prod..

[38] Li H., Xu C., Yuan Z., Wei Q. (2018). Synthesis of bio-based polyurethane foams with liquefied wheat straw: Process optimization. Biomass Bioenergy.

[39] Serrano L., Rincon E., García A., Rodríguez J., Briones R. (2020). Bio-degradable polyurethane foams produced by liquefied polyol from wheat straw biomass. Polymers.

[40] Xu C., Ferdosian F. (2017). Conversion of lignin into bio-based chemicals and materials, Ch. 8. Lignin-based polyurethane (PU) resins and foams. Green Chemistry and Sustainable Technology.

[41] Alinejad M., Henry C., Nikafshar S., Gondaliya A., Bagheri S., Chen N., Singh S.K., Hodge D.B., Nejad M. (2019). Lignin-based polyurethanes: Opportunities for bio-based foams, elastomers, coatings and adhesives. Polymers.

[42] Borges da Silva E.A., Zabkova M., Araujo J.D., Cateto C.A., Barreiro M.F., Belgacem M.N., Rodrigues A.E. (2009). An integrated process to produce vanillin and lignin-based polyurethanes from Kraft lignin. Chem. Eng. Res. Des..

[43] Pan X., Saddler J.N. (2013). Effect of replacing polyol by organosolv and kraft lignin on the property and structure of rigid polyurethane foam. Biotechnol. Biofuels.

[44] Huang X., De Hoop C.F., Xie J., Hse C.Y., Qi J., Hu T. (2017). Characterization of Biobased Polyurethane Foams Employing Lignin Fractionated from Microwave Liquefied Switchgrass. Int. J. Polym. Sci..

[45] Bernardini J., Cinelli P., Anguillesi I., Coltelli M.-B., Lazzeri A. (2015). Flexible polyurethane foams green production employing lignin or oxypropylated lignin. Eur. Polym. J..

[46] Muller L.C., Marx S., Vosloo H.C.M.J. (2017). Polyol preparation by liquefaction of technical lignins in crude glycerol. Renew. Mater..

[47] Nadji H., Bruzzese C., Belgacem M.N., Benaboura A., Gandini A. (2005). Oxypropylation of lignins and preparation of rigid polyurethane foams from the ensuing polyols. Macromol. Mater. Eng..

[48] Cateto C.A., Barreiro M.F., Rodrigues A.E., Belgacem M.N. (2009). Optimization study of lignin oxypropylation in view of the preparation of polyurethane rigid foams. Ind. Eng. Chem. Res..

[49] Cateto C.A., Barreiro M.F., Ottati C., Lopretti M., Rodrigues A.E., Belgacem M.N. (2014). Lignin-based rigid polyurethane foams with improved biodegradation. J. Cell. Plast..

[50] Berrima B., Mortha G., Boufi S., El Aloui E., Belgacem M.N. (2016). Oxypropylation of soda lignin: Characterization and application in polyurethane foams production. Cellul. Chem. Technol..

[51] Li H., Dun C., Jariwala H., Wang R., Cui P., Zhang H., Dai Q., Yang S., Zhang H. (2022). Improvement of bio-based polyurethane and its optimal application in controlled release fertilizer. J. Control. Release.

[52] Zhang Q., Lin X., Chen W., Zhang H., Han D. (2020). Modification of rigid polyurethane foams with the addition of nano-SiO_2_ or lignocellulosic biomass. Polymers.

[53] Baysal G., Aydın H., Hosgoren H., Uzan S., Karaer H. (2016). Improvement of synthesis and dielectric properties of polyurethane/Mt-QASs+ (novel synthesis). J. Polym. Environ..

[54] Moghaddam S.T., Naimi-Jamal M.R. (2019). Reinforced magnetic polyurethane rigid (PUR) foam nanocomposites and investigation of thermal, mechanical, and sound absorption properties. J. Thermoplast. Compos. Mater..

[55] Machado G., Leon S., Santos F., Lourega R., Dullius J., Mollmann M.E., Eichler P. (2016). Literature review on furfural production from lignocellulosic biomass. Nat. Resour..

[56] Nanni G., Heredia-Guerrero J.A., Paul U.C., Dante S., Caputo G., Canale C., Athanassiou A., Fragouli D., Bayer I.S. (2019). Poly(furfuryl alcohol)-polycaprolactone Blends. Polymers.

[57] Sun D., Sato S., Ueda W., Primo A., Garcia H., Corma A. (2016). Production of C4 and C5 alcohols from biomass-derived materials. Green Chem..

[58] Chen S., Wojcieszak R., Dumeignil F., Marceau E., Royer S. (2018). How catalysts and experimental conditions determine the selective hydroconversion of furfural and 5-hydroxymethylfurfural. Chem. Rev..

[59] Nakagawa Y., Tamura M., Tomishige K. (2013). Catalytic reduction of biomass-derived furanic compounds with hydrogen. ACS Catal..

[60] Nakagawa Y., Tamura M., Tomishige K. (2015). Catalytic conversions of furfural to pentanediols. Catal. Surv. Asia.

[61] Simeonov S.P., Ravutsov M.A., Mihovilovic M.D. (2019). Biorefinery via Achmatowicz rearrangement: Synthesis of pentane-1,2,5-triol from furfuryl alcohol. ChemSusChem.

[62] Simeonov S., Lazarova H., Marinova M., Popova M. (2019). Achmatowicz rearrangement enables hydrogenolysis-free gas-phase synthesis of pentane-1,2,5-triol from furfuryl alcohol. Green Chem..

[63] (2021). Rigid Cellular Plastics—Determination of Compression Properties.

[64] (2016). Standard Practice for Polyurethane Raw Materials: Polyurethane Foam Cup Test.

[65] (2006). Cellular Plastics and Rubbers—Determination of Apparent Density.

[66] (2016). Rigid Cellular Plastics—Determination of the Volume Percentage of Open Cells and of Closed Cells.

[67] (2001). Rigid Cellular Plastics—Determination of Water Absorption.

[68] (1986). Cellular Plastics, Rigid—Test for Dimensional Stability.

[69] (2016). Plastics—Biobased Content—Part 4: Determination of Biobased Mass Content.

[70] Jiao L., Xiao H., Wang Q., Sun J. (2013). Thermal degradation characteristics of rigid polyurethane foam and the volatile products analysis with TG-FTIR-MS. Polym. Degrad. Stab..

[71] Septevani A.A., Evans D.A.C., Chaleat C., Martin D.J., Annamalai P. (2015). K A systematic study substituting polyether polyol with palm kernel oil based polyester polyol in rigid polyurethane foam. Ind. Crops Prod..

[72] Gosz K., Kowalkowska-Zedler D., Haponiuk J., Piszczyk L. (2020). Liquefaction of alder wood as the source of renewable and sustainable polyols for preparation of polyurethane resins. Wood Sci. Technol..

